# A novel anxiety index for the rat behavior in the elevated plus-maze

**DOI:** 10.1186/1471-2202-14-S1-P326

**Published:** 2013-07-08

**Authors:** Rafael Arantes, Julian Tejada, Geraldine G Bosco, Silvio Morato, Antonio C Roque

**Affiliations:** 1 Departamento de Física, FFCLRP, Universidade de São Paulo, Ribeirão Preto, SP, 14040-901, Brazil; 2 Departamento de Matemática e Computação, FFCLRP, Universidade de São Paulo, Ribeirão Preto, SP, 14040-901, Brazil; 3 Departamento de Psicologia e Educação, FFCLRP, Universidade de São Paulo, Ribeirão Preto, SP, 14040-901, Brazil

## 

The elevated plus-maze (EPM) is a widely used anxiety animal model [1]. It consists of a plus-shaped maze elevated 50 cm from the floor. The maze is composed of two open arms and two closed arms crossing at right angles at a central area. The usual anxiety measures are the percentages of open or closed arm entries and the percentages of time spent on open or closed arms [2]. Recently, we have proposed a graph representation of the EPM in which the maze floor is divided into 21 squares that are treated as nodes of a directed graph [3], i.e. transitions between connected nodes can occur in both directions. Using the directed graph representation we have shown that the displacements of the rat in the EPM during a 5 min trial can be well modeled by a first-order homogeneous Markov chain [4]. Moreover, we have shown that the estimated stochastic matrix, which gives the transition probabilities between nodes, is sensitive to the pharmacological condition of the animal, i.e. if it belongs to the control group or was treated with an anxiogenic or anxiolytic drug [4]. The stochastic matrices for the different drug treatment types are interesting to compare different groups of rats but they are not practical as succinct indexes of their anxiety level. Here we propose a new anxiety index for the rat behavior in the EPM based on measures from the stochastic matrix.

The stochastic matrices were estimated from the recorded behaviors of 107 rats submitted to the EPM test under different pharmacological conditions. Twenty-five of the rats were control animals, 44 received anxiogenic drugs (pentylenetetrazol, 10, 20, and 30 mg/kg; semicarbazide, 20 mg/kg), and 38 received anxiolytic drugs (midazolam, 1 µg/kg; chlordiazepoxide, 5 mg/kg). From these matrices, the mean number of transitions until the rat reaches for the first time one of the nodes in the open or closed arms, starting from the central node at time 0, is calculated for all graph nodes. These mean values are called average absorption times of the nodes, which can be grouped in different ways. In this work, we considered the ratio of the average absorption time of a closed arm node to the average absorption time of the symmetrical node on the open arm. For high doses of anxiogenic drugs not all graph nodes are reached by the rat. These nodes were discarded from our calculations.

Our results show that for each pharmacological condition the ratios determined as explained above are approximately the same for any distance from the center. This invariance suggests that only one of the average absorption time ratios can be used to characterize the pharmacological condition of the animal. Based on this finding we propose that the average absorption time ratio can be used as an additional index to characterize the anxiety-related behavior of the rat in the EPM.
